# Overweight or Obesity Rate and Risk Factors in First-Episode and Drug-Naïve Patients with Major Depressive Disorder with Comorbid Abnormal Lipid Metabolism: A Large-Scale Cross-Sectional Study

**DOI:** 10.3390/metabo14010026

**Published:** 2023-12-30

**Authors:** Xiao Huang, Yuan Sun, Xiangyang Zhang

**Affiliations:** 1 Department of Anesthesiology, Beijing Chao-Yang Hospital, Capital Medical University, Beijing 100020, China; huanghuang94@yeah.net; 2 Department of Pharmacy, Peking University Third Hospital, Beijing 100191, China; sunny5106@163.com; 3 CAS Key Laboratory of Mental Health, Institute of Psychology, Beijing 100101, China; 4 Department of Psychology, University of Chinese Academy of Sciences, Beijing 101408, China

**Keywords:** overweight, obesity, major depressive disorder, abnormal lipid metabolism, rate

## Abstract

Overweight and obesity are frequent symptoms in patients with major depressive disorder (MDD) and abnormal lipid metabolism (ALM). There are no studies on the rate, risk factors, and underlying mechanisms of overweight/obesity in Chinese patients with MDD with comorbid ALM. The purpose of this study was to examine the rate of overweight/obesity and the associated risk factors among Chinese patients with MDD first-episode and drug-naïve (FEDN) with comorbid ALM. This study was a cross-sectional research work. A total of 1718 patients were enrolled. Their clinical and laboratory data were obtained. All participants were assessed with the 17-item Hamilton Depression Rating Scale (HAMD), the Hamilton Anxiety Rating Scale (HAMA), and the Positive and Negative Syndrome Scale (PANSS) positive subscale. The plasma total cholesterol (TC), low-density lipoprotein cholesterol (LDL-C), high-density lipoprotein cholesterol (HDL-C), triacylglycerols (TG), blood glucose concentrations, thyroid peroxidase antibody (A-TPO), thyoglubulin antibody (A-TG), thyroid-stimulating hormone (TSH), free thyoxine (FT4) and free triiodothyronine (FT3), and blood glucose concentrations were measured. ALM was identified as elevations in the plasma lipid values in this study. Of all the included subjects, the rate of ALM was 81.1%. The rate of obesity and overweight was 3.94% and 57.21%, respectively. Logistic regression analysis showed that TSH was the independent risk factor for overweight or obesity in MDD patients (adjusted OR = 1.158, 95%CI = 1.081–1.24, *p* < 0.001). The risk of developing overweight or obesity in MDD with ALM with comorbid TSH abnormalities was 2.176 times higher than those without TSH abnormalities (*p* < 0.001). Further linear regression showed TSH level (B = 0.1, t = 3.376, *p* = 0.001) and systolic blood pressure (B = 0.015, t = 2.351, *p* = 0.019) were risk factors for a higher body mass index (BMI). Our results demonstrate that being overweight is very frequent among patients with FEDN MDD with comorbid ALM but not obesity. TSH was the risk factor for overweight and obesity in MDD patients with comorbid ALM.

## 1. Introduction

Major depressive disorder (MDD) is recognized as one of the most prevalent mental disorders [1,2]. It frequently coexists with other chronic illnesses, and the presence of comorbid depression exacerbates health conditions compared with depression in isolation. Altered metabolic pathways and neurotransmitter concentrations are associated with MDD [3]. Both obesity and depression are pervasive conditions that significantly contribute to public health challenges [4,5,6]. In China, the estimated prevalence of overweight and obesity in adults (≥18 years) was 34.3% and 16.4%, respectively, from 2015 to 2019 [7]. Frigerio et al. highlighted multiple metabolic abnormalities in individuals with overweight and obesity [8], and being overweight is often linked to depressive symptoms [9].

The rates of overweight and obesity has been inconsistently reported in previous studies. Global estimates project that by 2030, about 38% of adults will be overweight, with an additional 20% classified as obese according to global estimates [10]. In the US, over 60% of adults are categorized as overweight or obese [11]. The prevalence of obesity in MDD ranges from 30% to 70% [12]. A meta-analysis by Rao et al. demonstrated that children and adolescents with obesity face a higher risk of MDD compared with their healthy counterparts [13]. A German study indicated a 1.3% prevalence of comorbid depression and obesity in men and 2.0% in women. Notably, low socioeconomic status and limited social support were associated with a higher prevalence of this comorbidity in women [14]. Existing data suggest the need for integrated treatment for depression and overweight/obesity [15]. The intricate interplay between the brain, fat cells, and gut could play a pivotal role [16]. Consequently, investigating the shared neurostructural associations of this common comorbidity is essential, yet data on Chinese or Asian patients are limited.

Dyslipidemia emerges as a significant player in various metabolic pathways. Lipids, including triglycerides (TG), phosphoglycerides, sterols, and lipids, constitute a diverse group of water-insoluble molecules. Abnormal lipid metabolism (ALM) and obesity frequently coexist in individuals with depression [17]. Wang et al. suggested that the rising incidence of obesity-related depression may be linked to metabolic health status [18]. Huang et al. identified a significant interaction between sedentary behavior and TG, increasing the risk of depression [19]. Individuals with MDD exhibited a higher incidence of hypertriglyceridemia than the controls [20]. Obesity-related dyslipidemia is characterized by low HDL-C levels and higher levels of TG-rich lipoproteins [21].

The rates of overweight and obesity in depressed patients have been inconsistently reported. Still, there is a lack of surveys on overweight and obesity in MDD with comorbid ALM for the Chinese mainland population. To address this gap, we conducted the first study to investigate the rates of overweight/obesity and risk factors in first-episode drug-naïve (FEND) MDD patients with comorbid ALM in the Chinese population. We enrolled a substantial number of FEND MDD patients (*n* = 1718) from the Chinese Han population, aiming to explore the rates of overweight/obesity and the associated risk factors.

## 2. Methods

### 2.1. Study Design and Subjects

In this cross-sectional study, the designated research unit was the First Hospital of Shanxi Medical University. Patients were recruited from the hospital after obtaining ethical approval, and informed consent was obtained from each patient.

Following the DSM-IV clinical interview, two psychiatrists confirmed the diagnosis of MDD. Enrollment was offered to those Han Chinese individuals aged between 18 and 60 years old who had not been treated with antidepressants, had experienced only one episode of MDD, suffered from the disease for less than two years, were neither pregnant nor nursing, and had no nervous system disorders or life-threatening illnesses.

**Figure 1:**
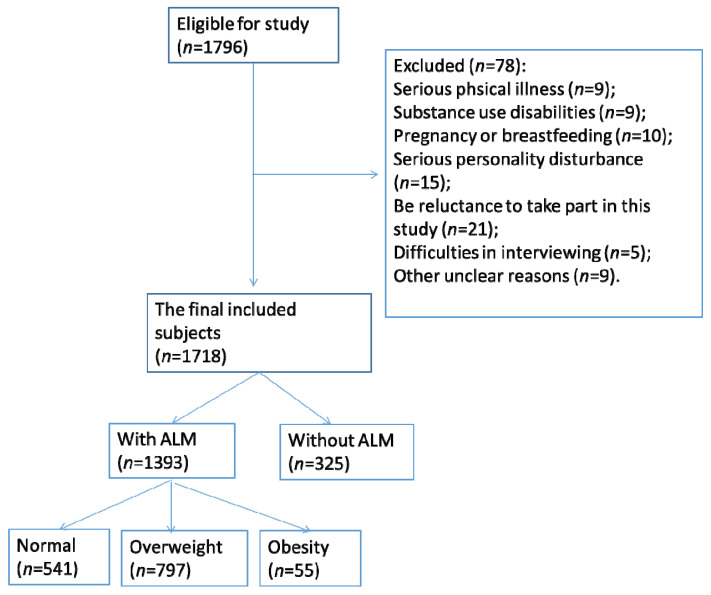
Flowchart of this study.

In addition, patients’ medical histories were examined, followed by a physical examination and laboratory examination. Those with serious physical or mental illnesses or a reluctance to provide formal consent were excluded. A total of 1718 subjects were included in the investigation, as illustrated in Figure 1.

### 2.2. Socio-Demographic Information Collection

Socio-demographic data were derived from a standard questionnaire, encompassing patient information such as sex, age, duration of illness, disease progression, education, and suicidal tendency. The questionnaire was administered by a trained research psychiatrist. Body mass index (BMI), a key metric for assessing obesity and overall health, was calculated based on body weight.

Body weight, measured with precision to 0.1 kg using an electronic scale (EB9003L, Guangdong Xiangshan Weighing Instrument Group Co., Ltd., Zhongshan, Guangdong, China), was calculated for the characterization of BMI. Patients were instructed to be barefoot and wear lightweight clothing for weight recording. According to the value of BMI values, eligible patients were categorized into three groups: normal weight, overweight group, and obese group, following the classification standards set by the Working Group on Obesity in China (WGOC) [22]. When BMI < 24 kg/m^2^, it was recorded as normal, between 24~28 kg/m^2^ was overweight, and ≥28 kg/m^2^ was obese.

Utilizing scales developed by Hamilton in 1959 and 1960, the Hamilton Anxiety Scale (HAMA) and Hamilton Depression Scale (HAMD) are widely employed for the clinical evaluation of anxiety and depression. In this study, patients were evaluated for depression and anxiety, with an additional assessment of the Positive and Negative Syndrome Scale (PANSS) positive subscale. The scales had a high reliability and validity in the Chinese population [23]. In our study, 24 was the threshold with or without MDD. HAMD-17 score ≥ 24 was an indicator of major depression [24,25].

Anxiety severity was quantified using the HAMA scale, where patients scoring above 29 were considered to have a severe anxiety disorder. Those scoring below this threshold were categorized as not having a severe anxiety disorder [26].

The severity of mental illness was described by the PANSS positive subscale [27]. Each option in the survey was assigned a score, increasing in severity from 1 to 7. In this study, patients with a scale score of 15 or greater were considered to have psychotic symptoms [28,29].

Before the investigation, two licensed psychiatrists received training on the above three scales. Trained psychiatrists were assigned to rate the scales before the start of the study. Both doctors scored patients by HAMA, HAMD, and PANSS, and the correlation coefficients between doctors were 0.84, 0.85, and 0.82, respectively.

### 2.3. Serum Assays

Patients were required to fast for half a day before obtaining intravenous blood samples. Following blood collection, comprehensive laboratory tests were conducted, encompassing assessments of the total cholesterol, high-density lipoprotein cholesterol, low-density lipoprotein cholesterol, triglyceride, and so on. Serum assays for thyroid peroxidase antibody (A-TPO), thyroglobulin antibody (A-TG), thyroid stimulating hormone (TSH), free thyroxine (FT4), and free triiodothyronine (FT3) performed using the Roche C6000 electrochemiluminescence immunoassay (Nanchang Roche Medical Equipment Co., Nanchang, China). In the present study, patients met the criteria for ALM if they exhibited abnormalities in the following four areas of abnormalities: TC greater than or equal to 5.20 mmol/L was considered a high TC level, TG greater than or equal to 1.70 mmol/L was considered a high TG level; LDL-C ≥ 3.40 mmol/L was considered high, and HDL-C < 1.00 mmol/L was considered a low high-density lipoprotein cholesterol level [30].

### 2.4. Statistics

To check the normal distribution of the data, the Q−Q plot and Shapiro−Wilk test were applied. Homogeneity of variances was performed by Levene’s test. Distributed continuous normal values were shown as mean ± standard deviation (SD), non-normally distributed items were displayed as the median (quartiles), and categorical measures were described as numbers and percentages. In this study, the mean ± standard deviation (SD) were used as a normally distributed continuous value, the median (quartiles) was a non-normally distributed item, and the numbers and percentages were categorical measures. Categorical data were treated with the χ^2^ test. ANOVA or Kruskal−Wallis rank test were conducted to compare differences between BMI values considering normality and equality of variance tests. For multiple comparisons, we selected Bonferroni correction. Post hoc analysis was carried out for a two-to-two comparison after ANOVA. The dependent variable was overweight or obese and the independent variable exhibiting significant differences in the previous analysis were used for binary logic regression analysis. In order to quantify the strength, we evaluated the odds ratio (OR) and the 95% confidence interval (95%CI). Finally, to analyze the risk factors of high BMI in patients with MDD complicated with ALM, we used regression analysis. Covariates with a variance inflation factor (VIF) > 2.5 were excluded from the regression. All of the analyses were completed in SPSS (Version 26.0). We used two-sided statistical methods with a *p*-value less than 0.05.

## 3. Results

### 3.1. Rate of Overweight and Obesity in MDD Patients with Comorbid ALM

A total of 1718 subjects were recruited based on the inclusion criteria. The rate of ALM was 81.1% (1393/1718). Table 1 compares the demographic parameters and clinical variables between patients with ALM and patients without ALM. The results showed that the two groups differed significantly on many factors. Compared with patients without ALM, patients with ALM were older (F = 4.859, *p* = 0.028), had a later age of onset (F = 4.679, *p* = 0.031) and a longer duration of disease (F = −3.138, *p* = 0.002), higher BMI (F = 9.734, *p* = 0.002), higher HAMA score (F = 37.843, *p* < 0.001), higher HAMD score (F = 172.701, *p* < 0.001), and higher PANSS score (F = −7.599, *p* < 0.001). There was no significant difference in terms of gender, educational level, and marital status (*p* > 0.05). However, after Bonferroni correction, no significant difference was found in age, age at onset, and BMI classification. The rate of overweight in MDD patients with ALM and without ALM was 57.2% and 50.8%, respectively. The rate of obesity in MDD patients with ALM and without ALM was 3.9% and 2.8%, respectively (Table 1). Logistics regression showed that ALM was a risk factor for overweight (OR = 1.348, 95%CI = 1.054–1.725, *p* = 0.017). After adjusting for sex and age, ALM was still a risk factor of overweight (OR = 1.323, 95%CI = 1.033–1.694, *p* = 0.026). However, ALM was not a risk factor for obesity (OR = 1.706, 95%CI = 0.824–3.531, *p* = 0.15).

### 3.2. Demographic and Clinical Variables in Overweight and Obesity in MDD Patients with Comorbid ALM

Table 2 compares the demographic and clinical data of patients with or without overweight and obesity in MDD with comorbid ALM. The results show that there was a significant difference in age, age at onset, disease duration, marital status, HDL-C level, TSH level, TC level, LDL-C level, blood glucose concentration, systolic and diastolic blood pressure, and severe anxiety (all *p* < 0.05) (Table 2).

Table 3 demonstrates the results of the logistic regression analysis. We included the disease duration, marriage, TSH, blood glucose concentration, TC level, HDL-C level, LDL-C level, systolic blood pressure, and severe anxiety in the regression. The results showed that the only variable independently associated features with overweight and obesity in MDD patients with comorbid ALM was the TSH level (after adjusting for age, sex, educational level, anxiety, and exhibiting psychotic symptoms: OR = 1.158, 95%CI = 1.081–1.24, *p* < 0.001) (Table 3). The risk of developing overweight or obesity in MDD with ALM with comorbid TSH abnormalities (TSH ≥ 4.5 mIU/L) was 2.176 times higher in MDD patients with ALM with comorbid TSH abnormalities (TSH < 4.5 mIU/L) after being adjusted for age, sex, educational level, anxiety, and exhibiting psychotic symptoms (95%CI = 1.726–2.743, *p* < 0.001) than in patients without ALM (Table 4). Further linear regression showed that the TSH level (B = 0.1, t = 3.376, *p* = 0.001) and systolic blood pressure (B = 0.015, t = 2.351, *p* = 0.019) were risk factors for a higher BMI.

## 4. Discussion

Numerous studies have explored the link between obesity and ALM due to its etiological and therapeutic implications. However, none have examined the proportions and risk factors of overweight and obesity in MDD with comorbid ALM.

A primary finding of our study was that the rates of overweight and obesity in MDD with comorbid ALM were 57.2% and 3.9%, respectively. Rates of overweight and obesity in depression vary in the literature. For instance, Luo et al.’s extensive longitudinal study in China reported overweight and obese rates of 35.03 and 16.84% for women and 28.07 and 9.26% for men, respectively [31]. Another large study of women aged 40 to 65 found obesity rates of 25.4% for those without depressive symptoms and 57.8% for those with moderate to severe depression [32]. Our study’s overweight rate was significantly higher than those mentioned above, but the obesity rate was lower. This divergence may be attributed to differences in subjects’ age and location. Additionally, our study included MDD patients, while the aforementioned studies focused on individuals with depressive symptoms. Li et al. reported a 24.8% overweight rate and a 9.9% obesity prevalence in MDD patients from urban southern China, further emphasizing the diversity in reporting rates [33].

Our study also revealed that overweight or obese MDD patients had higher levels of TG, TC, and LDL-C levels compared with those who were not overweight or obese, consistent with previous findings [34]. Obesity heightens cardiovascular risk by elevating fasting plasma triglycerides, high LDL-C levels, and low HDL-C levels. Insulin resistance in peripheral tissues may contribute to the link between obesity, metabolic syndrome, and dyslipidemia [35]. Long non-coding RNA (lncRNA) plays a crucial role in regulating abnormal metabolism in obesity syndrome [36]. Du et al. indicated a correlation between centrally obese participants and hypothyroidism and depression, with BMI values strongly correlated with lipid levels [37]. The AMPK/mTOR signaling pathway may be involved in lipid metabolism or promote autophagy in obesity depression [38]. Experiencing overweight, obesity, and other metabolic abnormalities are significant risk factors for psychiatric disorders like MDD, acknowledging the potential influence of psychotropic drugs [39]. While our study did not delve into the effects of psychotropic drugs, understanding the exact mechanisms requires further exploration.

Another significant finding is that TSH was identified as a risk factor for overweight and obesity in MDD with ALM. The risk of being overweight or obese was twice as high in MDD patients with comorbid thyroid abnormalities than in those with normal thyroid function. Numerous studies have affirmed the crucial role of thyroid function in overweight and obesity patients. For instance, Drivsholm et al. noted higher TSH concentrations and increased blood pressure in obese patients [40]. Laclaustra et al. reported a correlation between a higher index of thyroid hormone resistance and obesity and metabolic syndrome [41]. Thyroid hormones influence energy expenditure by regulating cellular respiration and thermogenesis. Subclinical hypothyroidism can alter the basal metabolic rate and increase BMI, while obesity can affect thyroid function through various mechanisms [42]. Only one study has explored the association between thyroid dysfunction and BMI in Chinese patients with FEND MDD, indicating higher TSH levels in overweight/obese patients [43]. However, the complex reasons behind this association remain poorly understood. This underscores the need for future prospective causality studies in this area.

As both depression and metabolic syndrome (MetS) are risk factors for cardiovascular illness, their relationship has received considerable interest in recent years. For example, Zhang et al. showed that depression was a modifier contributing to MetS and its components (waist circumference, hypertension, FBG, TG, and HDL-C) in a Mendelian randomization [44]. Moradi et al. found that that depression increased the risk of MetS by 48% in a meta-analysis of observational studies [45]. A cross-sectional study by Moreira et al. in young adults aged 24 to 30 years found a higher prevalence of MetS in patients with depression and anhedonia; furthermore, patients with depression and hedonism had significantly higher levels of glucose, triglycerides, TC, and LDL-C, while their levels of HDL-C were lower [46]. Pimenta et al. conducted a large prospective cohort study, and found that there was no association between depression and the incidence of MetS at baseline, but comorbid depression after 2 years of follow-up was strongly associated with an increased risk of new-onset MetS [47]. All of the above findings suggest that depressive disorders may be a modifier contributing to the heterogeneity of metabolic syndrome. Our study strengthens the evidence that there is a close association between depression and MetS and its components. Several shared pathways, including inflammation, the hypothalamic−pituitary−adrenal axis, oxidative stress, coronary artery disease, etc., may play an important role in the link between depression and MetS [48], but the underlying mechanisms need to be explored in future studies.

## 5. Limitation

Our study had several limitations. Firstly, the participants were exclusively Han Chinese outpatients, necessitating validation in diverse populations. The cross-sectional design precluded establishing causality, warranting future prospective cohort studies. Unconsidered risk factors in our study included income, activity levels, parental obesity, and other comorbidities. Dietary habits, lifestyle, substance use, and chronic diseases associated with obesity were not explored.

Additionally, the study did not investigate the lipid metabolism function of patients before depression onset, making it challenging to determine whether ALM preceded depression or resulted from it. Furthermore, the lipid levels were measured only once, introducing potential random errors. Lastly, our study focused on patients with numerical ALM and may not generalize to the broader dyslipidemia population.

## 6. Conclusions

In conclusion, our study revealed a 57.2% and 3.9% rate of overweight and obesity, respectively, in first-episode and drug-naïve MDD patients with comorbid ALM. Notably, TSH emerged as a risk factor for overweight and obesity in MDD with comorbid ALM. Given the high prevalence of comorbid overweight/obesity in MDD patients, thyroid function should be carefully considered in this population.

## Figures and Tables

**Table 1:** Demographic and clinical variables in MDD with ALM and without ALM.

	With ALM (*n* = 1393)	Without ALM (*n* = 325)	*p*-Value
Age, year, mean ± SD ^a^	35.2 (12.5)	33.5 (11.9)	0.028
Age of onset, year, mean ± SD ^a^	35 (12.4)	33.3 (11.9)	0.031
Duration of illness, year, mean ± SD ^a^	6.5 (4.11)	5 (4.8)	0.002
Sex, n (%) ^c^			0.255
Male	468 (33.6)	120 (36.9)	
Female	925 (66.4)	205 (63.1)	
Education, n (%) ^c^			0.717
Junior high school	342 (24.6)	71 (21.8)	
Senior high school	615 (44.1)	145 (44.6)	
College	358 (25.7)	91 (28)	
Postgraduate	78 (5.6)	18 (5.5)	
Marry status, n (%) ^c^	999 (71.7)	217 (66.8)	0.077
BMI, kg/m^2^, mean ± SD ^a^	24.4 (2)	24.1 (1.7)	0.002
BMI grouping, n (%) ^c^			0.034
Normal	541 (38.8)	151 (46.5)	
Overweight	797 (57.2)	165 (50.8)	
Obesity	55 (3.9)	9 (2.8)	
HAMD, mean ± SD ^a^	30.7 (2.8)	28.5 (2.7)	<0.001
HAMA, mean ± SD ^a^	21(3.5)	19.7 (3.3)	<0.001
PANSS, median [IQR] ^b^	7 (7.9)	7 (7.7)	<0.001

Note: Data expressed as mean ± SD, median (interquartile range), or percentage. BMI: HAMD: Hamilton Rating Scale for Depression. HAMA: Hamilton Anxiety Scale. PANSS: Positive and Negative Syndrome Scale. ^a^ Analysis of covariance (ANCOVA) for distributed variables. ^b^ non-parametric Mann−Whitney test for non-normally distributed variables. ^c^ Chi-square test for categorical variables.

**Table 2:** Demographic and clinical variables in overweight and obesity in MDD patients with comorbid ALM.

	Normal (*n* = 541)	Overweight (*n* = 797)	Obesity (*n* = 55)	*p*-Value	*p*-Value		
					Normal vs. Overweight	Normal vs. Obesity	Overweight vs. Obesity
Age, year, median [IQR] ^b^	32 (21, 45)	36 (25, 46)	33 (26, 43)	0.011	0.003	0.997	0.349
Duration of illness, year, median [IQR] ^b^	5 (3, 8)	6 (3, 8.5)	5 (3, 9)	0.014	0.004	0.705	0.822
Age of onset, year, median [IQR] ^b^	32 (21, 45)	36 (25, 46)	32 (26, 42)	0.011	0.003	0.998	0.343
Sex, n (%) ^c^				0.165	0.989	0.066	0.062
*Male*	179 (33.1)	264 (33.1)	25 (45.5)				
*Female*	362 (66.9)	533 (66.9)	30 (54.5)				
Education, n (%) ^c^				0.337	0.476	0.405	0.151
Junior high school	125 (23.1)	205 (25.7)	12 (21.8)				
Senior high school	239 (44.2)	357 (44.8)	19 (34.5)				
College	143 (26.4)	196 (24.6)	19 (34.5)				
Postgraduate	34 (6.3)	39 (4.9)	5 (9.1)				
Marry status, n (%) ^c^	365 (67.5)	592 (74.3)	42 (76.4)	0.019	0.007	0.177	0.732
BMI, kg/m^2^, median [IQR] ^b^	23.1 (22.1, 23.5)	25.4 (24.5, 26.2)	28.3 (28.1, 28.6)	<0.001	<0.001	<0.001	<0.001
HAMD, mean ± SD ^a^	30.7 (2.9)	30.7 (2.8)	31.4 (2.4)	0.255	0.98	0.107	0.104
HAMA, mean ± SD ^a^	21 (3.2)	21 (3.6)	21.2 (3.4)	0.975	0.967	0.837	0.822
PANSS, median [IQR] ^b^	7 (7, 8)	7 (7, 9)	7 (7, 10)	0.436	0.608	0.2	0.285
CGI, median [IQR] ^b^	6 (5, 7)	6 (5, 7)	6 (6, 7)	0.479	0.651	0.228	0.3
A-TG, IU/Ml, median [IQR] ^b^	22.3 (15.1, 49.8)	21.7 (14.4, 49)	22.2 (14.5, 52.5)	0.327	0.135	0.715	0.814
A-TPO, IU/Ml, median [IQR] ^b^	18.9 (12.7, 35.9)	16.7 (12, 36.2)	17.6 (12.2, 32.7)	0.118	0.399	0.605	0.769
TSH, mIU/L, mean ± SD ^a^	4.9 (2.7)	5.7 (2.4)	6.4 (1.6)	<0.001	<0.001	<0.001	0.063
FT3, pmol/L, mean ± SD ^a^	4.9 (0.7)	4.9 (0.7)	4.9 (0.7)	0.454	0.241	0.87	0.526
FT4, pmol/L, median [IQR] ^b^	16.4 (14.5, 18.8)	16.5 (14.5, 18.6)	17.6 (14.2, 19.5)	0.154	0.648	0.085	0.053
Blood glucose, mean ± SD ^a^	5.4 (0.6)	5.5 (0.7)	5.5 (0.6)	0.001	<0.001	0.109	0.882
TC, mmol/L, mean ± SD ^a^	5.4 (1.1)	5.5 (1.1)	5.8 (0.8)	0.023	0.369	0.006	0.015
LDL-C, mmol/L, mean ± SD ^a^	3.1 (0.9)	3.1 (0.9)	3.5 (1.1)	0.005	0.857	0.002	0.001
HDL-C, mmol/L, median [IQR] ^b^	1.23 (0.98, 1.44)	1.21 (0.95, 1.37)	1.22 (0.9, 1.4)	0.049	0.015	0.827	0.926
TG, mmol/L, median [IQR] ^b^	2.2 (1.6, 2.9)	2.3 (1.8, 2.9)	2.3 (1.4, 2.9)	0.54	0.265	0.761	0.971
SBP, mmHg, mean ± SD ^a^	118.5 (11.4)	121.4 (10.4)	123.4 (8.4)	<0.001	<0.001	0.001	0.169
DBP, mmHg, mean ± SD ^a^	75.7 (7)	76.7 (6.7)	76.7 (6.4)	0.02	0.006	0.272	0.992
Suicide attempt, n (%) ^c^	124 (22.9)	164 (20.6)	14 (25.5)	0.467	0.306	0.671	0.389
Anxiety, n (%) ^c^	55 (10.2)	116 (14.6)	9 (16.4)	0.047	0.018	0.157	0.714
Exhibiting psychotic symptoms, n (%) ^c^	54 (10.0)	96 (12)	8 (14.5)	0.377	0.24	0.291	0.584

Note: Data expressed as mean ± SD, median (interquartile range), or percentage. MDD: major depressive disorder; ALM: abnormal lipid metabolism; BMI: body mass index; HAMD: Hamilton Rating Scale for Depression; HAMA: Hamilton Anxiety Scale; PANSS: Positive and Negative Syndrome Scale; CGI: clinical global impression; A-TG: anti-thyroglobulin; A-TPO thyroid peroxidases antibody; TSH: thyroid stimulating hormone; FT3: free triiodothyronine; FT4: free thyroxine; TC: total cholesterol; LDL-C: low-density lipoprotein cholesterol; HDL-C: high-density lipoprotein cholesterol; TG: triacylglycerols; SBP: systolic blood pressure; DBP: diastolic blood pressure. ^a^ Analysis of covariance (ANCOVA) for distributed variables. ^b^ non-parametric Mann−Whitney test for non-normally distributed variables. ^c^ Chi-square test for categorical variables.

**Table 3:** The risk factors of overweight or obesity in MDD patients with ALM.

Variable	Unadjusted Model		Model Ⅰ		Model Ⅱ	
	OR (95%CI)	*p*-Value	OR (95%CI)	*p*-Value	OR (95%CI)	*p*-Value
Duration of illness	1.007 (0.983–1.032)	0.573	1.007 (0.982–1.032)	0.611	1.006 (0.981–1.031)	0.667
TSH	1.156 (1.083–1.233)	<0.001	1.157 (1.083–1.236)	<0.001	1.158 (1.081–1.24)	<0.001
Blood glucose	1.121 (0.923–1.361)	0.249	1.124 (0.926–1.365)	0.238	1.125 (0.927–1.367)	0.233
TC	0.879 (0.767–1.008)	0.064	0.879 (0.767–1.008)	0.064	0.878 (0.766–1.007)	0.062
HDL-C	0.942 (0.633–1.401)	0.768	0.941 (0.632–1.399)	0.763	0.949 (0.638–1.413)	0.798
LDL-C	0.905 (0.77–1.052)	0.193	0.905 (0.78–1.052)	0.194	0.905 (0.779–1.051)	0.191
SBP	1.009 (0.995–1.022)	0.21	1.008 (0.993–1.023)	0.292	1.008 (0.993–1.023)	0.299
Married	1.269 (0.964–1.672)	0.09	1.262 (0.913–1.746)	0.159	1.25 (0.903–1.731)	0.179

Note: The risk factors of overweight or obesity in MDD patients with ALM. Dependent variable: overweight or obesity; independent variables: duration of illness, TSH, blood glucose, TC, HDL-C, LDL-C, SBP, and married; TSH: thyroid stimulating hormone; MDD: major depressive disorder; ALM: abnormal lipid metabolism; TC: total cholesterol; HDL-C: high-density lipoprotein cholesterol; LDL-C: low-density lipoprotein cholesterol; SBP: systolic blood pressure; Model Ⅰ adjusted for age and sex; Model Ⅱ adjusted for age, sex, educational level, anxiety, and exhibiting psychotic symptoms.

**Table 4:** TSH level as the risk factors of overweight or obesity in MDD patients with ALM.

TSH Level (mIU/L)	Unadjusted Model		Model Ⅰ		Model Ⅱ	
	OR (95%CI)	*p* Value	OR (95%CI)	*p* Value	OR (95%CI)	*p* Value
<4.5	Reference					
≥4.5	2.332 (1.862–2.921)	<0.001	2.213 (1.76–2.783)	<0.001	2.176 (1.726–2.743)	<0.001
<2.1	Reference					
2.1–3.55	3.951 (2.721–5.736)	<0.001	3.913 (2.693–5.687)	<0.001	3.845 (2.635–5.612)	<0.001
3.56–4.49	2.981 (1.854–4.794)	<0.001	3.042 (1.888–4.899)	<0.001	2.958 (1.825–4.792)	<0.001
≥4.5	1.59 (1.025–2.469)	0.039	1.598 (1.028–2.484)	0.037	1.596 (1.027–2.482)	0.038

Note: TSH level as the risk factors of overweight or obesity in MDD patients with ALM. Dependent variable: overweight or obesity; independent variable: TSH level. MDD: major depressive disorder; ALM: abnormal lipid metabolism; TSH: thyroid-stimulating hormone; Model Ⅰ adjusted for age and sex; Model Ⅱ adjusted for age, sex, educational level, anxiety, and exhibiting psychotic symptoms.

## Data Availability

The data presented in this study are available on request from the corresponding author. The data are not publicly available due to privacy.
